# Altered temporal dynamics of brain activity in patients with generalized tonic-clonic seizures

**DOI:** 10.1371/journal.pone.0219904

**Published:** 2019-07-17

**Authors:** Honglei Liu, Wenling Li, Mingjuan Zhao, Jie Wu, Jing Wu, Jiankai Yang, Baohua Jiao

**Affiliations:** 1 Department of Neurosurgery, The Second Hospital of Hebei Medical University, Shijiazhuang, P.R. China; 2 Department of Neurosurgery, Shijiazhuang the Third Hospital, Shijiazhuang, P.R. China; 3 Medical Imaging Department, Hebei General Hospital, Shijiazhuang, P.R. China; University Paris 6, FRANCE

## Abstract

Generalized seizures engage bilateral networks from their onset at a low temporal scale. Previous studies findings have demonstrated focal/local brain activity abnormalities in the patients with generalized tonic-clonic seizures (GTCS). Resting state functional magnetic resonance imaging (fMRI) allows the detection of aberrant spontaneous brain activity in GTCS. Little is known, however, about alterations of dynamics (temporal variability) of spontaneous brain activity. It also remains unclear whether temporal variability of spontaneous brain activity is associated with disease severity. To address these questions, the current study assessed patients with GTCS (n = 35), and age- and sex-matched healthy controls (HCs, n = 33) who underwent resting state fMRI. We first assessed the dynamics of spontaneous brain activity using dynamic amplitude of low-frequency fluctuation (dALFF). Furthermore, the temporal variability of brain activity was quantified as the variance of dALFF across sliding window. Compared to HCs, patients with GTCS showed hyper-temporal variability of dALFF in parts of the default mode network, whereas they showed hypo-temporal variability in the somatomotor cortex. Furthermore, dynamic ALFF in the subgenual anterior cingulate cortex was positively correlated with duration of disease, indicating that disease severity is associated with excessive variability. These results suggest both an excessive variability and excessive stability in patients with GTCS. Overall, the current findings from brain activity dynamics contribute to our understanding of the pathophysiological mechanisms of generalized seizure.

## Introduction

Genetic generalized epilepsy (GGE), characterized by generalized spike-and slow waves, polyspikes, and polyspikes and waves on scalp electroencephalography (EEG) comprises a group of epileptic disorders [1]. Generalized tonic-clonic seizures (GTCS) is one of GGE subtype [2]. High myoelectric contamination on ictal scalp EEG and unseen epileptogenic structural abnormalities on magnetic resonance imaging (MRI) have so-far hindered a full understanding of the pathological mechanisms of GTCS [3].

Mounting evidence from multimodal brain connectivity-based studies suggests that GTCS involves a disruption in the topological organization of brain structural and functional networks [3,4]. Although GSWDs are highly synchronized, the thalamocortical network does not demonstrate abnormal functional connectivity as assessed by resting-state functional MRI (rs-fMRI) in patients with GGE including GTCS, juvenile absence epilepsy, eyelid myoclonia with absences, juvenile myoclonic epilepsy, and absences epilepsy [5]. Inconsistent with this finding, however, Ji et al. have demonstrated that patients with GTCS-only exhibit a wide range increase in functional connectivity in four thalamocortical networks [6]. These inconsistent findings are likely due to the inclusion of different subtypes of GGE patients in the two studies. Subsequently, combining anatomical connectivity derived from diffusion-weighted images (DTI)-tractography and functional connectivity, Ji et al. further found that both anatomical and functional connectivity between the bilateral anterior cingulate cortices (ACC) were increased. This suggests that the commissural fiber bundle in the genu of the corpus callosum may be a promising potential target for surgical treatment in intractable GTCS [7]. More strikingly, using graph theoretical analysis, Zhang et al. found that the patients with GTCS-only exhibited disrupted topological organization in large-scale brain functional (temporal correlation derived from rs-fMRI) and structural (derived from DTI tractography) networks. Specifically, Zhang et al. calculated the connection density (i.e., number of connections) between end-nodes in structural network [3], and found that the degree of coupling (spatial correlation) between structural and functional networks was decreased in GTCS [3]. Taken together, GTCS can been considered a brain network disorder rather than a single source pathology [8].

Although generalized seizures engage in bilateral networks from onset at a low temporal scale, focal functional abnormalities also emerge, suggesting locally and selectively impaired brain function in GTCS [9]. Furthermore, the amplitude of low-frequency fluctuation (ALFF) of rs-fMRI signals has served as an informative available tool to describe local brain activity [10]. The ALFF is thought to represent a meaningful relationship between the brain’s cerebral glucose metabolism [11] and morphology [12]. Generally, the ALFF describes the amplitude characteristics of local brain activity, and shows higher test-retest reliability than the other measures of functional connectivity or functional connectivity density in rs-fMRI [13,14]. ALFF thus provides a neuromarker to highlight brain regions with altered amplitudes in GTCS [15,16].

The epileptic brain has additional dynamic features, such as the paroxysmal occurrence of seizures [17]. The brain’s dynome (i.e., a map of its dynamic functional connectivity and dynamic brain activity) determines its temporal variability [18,19], characterizing aspects of a neural system’s functional capacity [20,21]. Specifically, Liao et al. for the first time found a complex transitions of functional network topology using the method of dynamic functional connectivity, implicating adaptive reconfiguration of functional brain networks in absence seizures [22]. More recently, functional connectivity analyses within the frontal, parietal and occipital cortices have revealed significantly reduced synchrony during the minute prior to discharge onset, suggesting that existence of a state of predisposition to generalized seizure [23]. Despite these observations, there is still a lack of information regarding the dynamics of brain activity in GTCS.

In the present study, we sought to investigate whether patients with GTCS-only show altered temporal dynamics of spontaneous brain activity at rest using a novel dynamic ALFF (dALFF). The dALFF quantifies the temporal variability of ALFF over time to track time-variant of brain activity [24,25]. Furthermore, we also sought to explore whether altered dALFF correlated with the clinical variables of patients with GTCS.

## Materials and methods

### Participants

This work was approved by the local Ethics Committee of Hebei General Hospital. Written informed consents were obtained from all participants. A total of 40 patients diagnosed as seizure types of GTCS only, according to the International League Against Epilepsy criteria, were recruited. The inclusion criteria were as follows: i) manifestation of typical symptoms of GTCS: the main symptoms are generalized tonic-clonic seizures, accompanied by a loss of consciousness; ii) presence of GSWDs: the generalized spike-and slow waves in interictcal discharge and generalized EEG onset during ictal phase; iii) no history of addiction; and iv) no structural abnormalities observed in MRI results. The exclusion criteria were as follows: i) falling asleep during rs-fMRI scanning; and ii) head motion exceeding 3 mm or 3°. In the end, a total of 35 patients with GTCS (14 females; age [mean ± standard deviation (SD)]: 29.00 ± 8.67 years) were included for further analyses. Demographic and clinical information of patients are shown in Table 1. Twenty-eight patients were undergoing treatment with anti-epileptic drugs, including valproate, phenytoin, carbamazepine, lamotrigine, and topiramate (Table 2).

**Table 1:** Participant demographic and clinical information.

Demographics	GTCS	HCs	Statistical	Evaluation
Group size (n)	35	33	N.A.	N.A.
Handedness (left/right)	0/35	0/33	N.A.	N.A.
Sex (male/female)	21/14	18/17	χ^2^ = 1.44	p = 0.23
Age (years)	29.00 ± 8.67	29.88 ± 6.98	U = 477.5	p = 0.22
Illness duration (months)	53.71 ± 43.14	N.A.	N.A.	N.A.

Values are mean ± SD.

**Table 2:** Demographic data of GTCS patients.

Patient	Sex	Age (y)	Duration (m)	Antiepileptic drugs	EEG ERR(Hz)
1	M	21	60	VPA	10–11→3–4
2	F	32	120	TCHM	10–11→3–4
3	M	21	5	None	10–11→3–4
4	M	22	108	VPA/LEV	12–13→2–3
5	F	37	48	LTG	10–11→3–4
6	M	25	14	None	10–11→3–4
7	F	22	25	LEV	13–14→3–4
8	M	47	16	TCHM	10–11→3–4
9	M	26	16	None	10–11→5–6
10	M	41	71	VPA/LTG	12–13→3–4
11	M	35	18	VPA	10–11→3–4
12	F	50	70	LEV	14–15→3–4
13	F	29	6	None	12–13→2–3
14	M	20	88	TCHM/VPA/LTG	12–13→3–4
15	F	22	15	LTG	10–11→3–4
16	F	24	10	TCHM/LTG	10–11→4–5
17	M	22	153	VPA/LEV	13–14→3–4
18	F	27	81	LTG	13–14→3–4
19	M	28	32	VPA	12–13→3–4
20	M	39	23	VPA	10–11→2–3
21	F	21	38	None	10–11→3–4
22	M	29	17	None	12–13→3–4
23	M	23	49	VPA	10–11→3–4
24	M	23	7	TCHM	10–11→3–4
25	M	23	137	VPA	14–15→5–6
26	M	33	45	LEV/VPA	12–13→3–4
27	M	48	4	None	10–11→3–4
28	F	38	36	LTG	14–15→3–4
29	F	41	76	LTG	10–11→5–6
30	M	25	98	VPA	12–13→2–3
31	M	20	95	VPA/LTG	10–11→3–4
32	F	31	60	LTG	10–11→3–4
33	F	23	117	LEV/LTG	10–11→2–3
34	M	25	114	VPA	13–14→3–4
35	F	22	8	TCHM/LTG	10–11→3–4

Abbreviations: m: month, y: year; VPA: valproate; TCHM: traditional Chinese herb medicine; LTG: lamotrigine; LEV: Levetiracetam; ERR: epileptic recruiting rhythm

Thirty-five sex- and age-matched healthy control (HCs) were recruited. The inclusion criteria were as follows: i) no history of neurological or psychiatric disorders; and ii) no structural abnormalities observed in MRI results. The exclusion criteria were as follows: i) falling asleep during resting-state fMRI scanning; and ii) head motion exceeding 3 mm or 3°. Two out of 35 HCs were excluded due to excessive head motion during rs-fMRI scanning. In the end, 33 HCs (18 females; age (mean ± SD): 29.88 ± 6.98 years) were included (Table 1).

### Data acquisition

The structural and functional images were acquired on a 3.0-T GE-Signa MRI scanner (EXCITE, General Electric, Milwaukee, USA) at the Hebei General Hospital. The 3D T1 imaging parameters were as follows: repetition time/echo time (TR/TE) = 2300/2.98 ms, matrix = 256 × 256, flip angle = 9°, voxel size = 1 × 1 × 1 mm^3^, 176 axial slices without interslice gap. Functional imaging parameters were as follows: TR/TE = 2000/30 ms, matrix = 64 × 64, flip angle = 90°, voxel size = 3.74 × 3.75 × 4 mm^3^, 31 slices without interslice gap. For each participant, a total of 240 volumes (480 s) of functional images were obtained. Subject were instructed to rest with their eyes closed, and were told not to think of anything in particular [26,27]. After each scanning session, the subjects were tested on whether they were asleep or awake by vocal communication [25]. Subjects were also asked directly if they had fallen asleep at the end of scanning.

### Data preprocessing

The imaging data were preprocessed using DPARSF (v4.3, http://www.restfmri.net) and SPM12 (http://www.fil.ion.ucl.ac.uk/spm) toolkits. The first 10 volumes (20 s) of rs-fMRI imaging were discarded. The remaining images were corrected for temporal differences and head motion. Five patients and two HCs were excluded due to excessive head motion at 3 mm translation or 3° rotation. The 3D T1 images were co-registered to the mean functional image of each participant. The co-registered 3D T1 images were then segmented as gray-matter, white-matter (WM) and cerebrospinal fluid (CSF), followed by normalization to Montreal Neurologic Institute (MNI) space. Next, functional images were also normalized to MNI space by DARTEL, and resized at a voxel size of 3 × 3 × 3 mm^3^. Subsequently, several spurious variances, including Friston 24 head motion parameters, as well as CSF and WM signals, were regressed out. To strictly control for head motion, we performed scrubbing of the functional images, allowing undesirable points to be regressed out. The undesirable points were defined as a threshold of framewise-displacement (FD) > 0.5 mm, as well as the time points one-forward and two-back [24,28]. However, the mean FD did not differ between the two groups (two-sample t-test, T = 0.56, p = 0.29). Each undesirable bad point was considered as a separate regressor. Functional images were then spatially smoothed using an 8 mm full-width half-maximum isotropic Gaussian kernel. Finally, linear trends were removed and temporal band-pass filtering (0.01–0.1 Hz) was performed. Since rs-fMRI signals contributed to the greatest portion of the amplitude (i.e. power) distribution at low frequency fluctuations [10], as with previous studies [24,25], we used this as the frequency domain. Any scrubbing which altered the nature of the temporal structure necessary for ALFF analysis was not used [24].

### dALFF computation

A sliding window approach was used to compute the dALFF using DynamicBC toolbox (v2.0, www.restfmri.net/forum/DynamicBC) [29]. Window length is considered an important parameter in the sliding window approach. Based on previous studies [28,30], a window length of 50 TRs (100 s) was used to calculate the temporal variability of dALFF [24]. The time courses were composed of 230 TRs (460 s), and the window overlap was chosen as 0.9, in accordance with previous study [24]. Next, the whole-length time courses were separated into 39 windows for each subject, and we obtained the ALFF map for each window. To investigate the temporal variability of brain activity, we computed the coefficient of variation (CoV: SD/mean) of dALFF maps across windows. After CoV transformation, the map of each voxel was divided by the global mean value in order to normalize global effects (defined as mCoV images).

### Statistical analysis

Demographic and clinical parameters were compared between the patients with GTCS and HCs. Age and sex were analyzed using Mann-Whitney U test and a χ^2^ test, respectively.

To evaluate the temporal variability of ALFF at a group-level, we averaged mCoV images from patients and HCs, separately. To further investigate the difference in temporal variability of ALFF between these two groups, a two-sample t-test was performed in a voxel-wise manner using the general linear model (GLM) in SPM12. Considering that age and sex affect ALFF analysis [13,31], we entered these two variables into the GLM as covariates for analysis of covariance (ANCOVA) for a two-group comparison [32]. Considering the huge number of time comparisons (about 47636 voxels) in the fMRI volume, we attempted to diminish Type I errors (i.e. false positives). With respect to Type I errors, a family-wise error (FWE) correction based on Gaussian field theory (GRF) using various cluster size corrections is conceptually similar to a Bonferroni test [33]. The results we report here were set with a primary voxel-wise threshold of *p* < 0.01, which yielded a cluster-extent based threshold of *k* > 150; meaning a cluster of a minimum of 150 voxels can be consider to be significant (cluster-level *p* < 0.05, Gaussian Random Field (GRF) correction).

Finally, to determine whether the abnormal dALFF regions were correlated with the clinical variables, including duration of disease and time of onset, a Pearson’s correlation was performed for the patient group. We computed the Pearson’s correlation coefficient using the appropriate dALFF and disease duration values. We used a Bonferroni-corrected statistical significance level of *p* < 0.05.

## Results

### Clinical and demographic characteristics

Seven subjects (five GTCS patients and two HCs) were excluded as their head motion exceeded 3.0 mm translation or 3.0° rotation during rs-fMRI scanning. In total, 35 GTCS patients and 33 HCs were included for analysis. There was no significant difference in age (Mann-Whitney U test, *p* = 0.22) or gender (χ^2^ test, *p* = 0.23) between the patients and HCs.

### Group differences of dALFF

**Fig 1:**
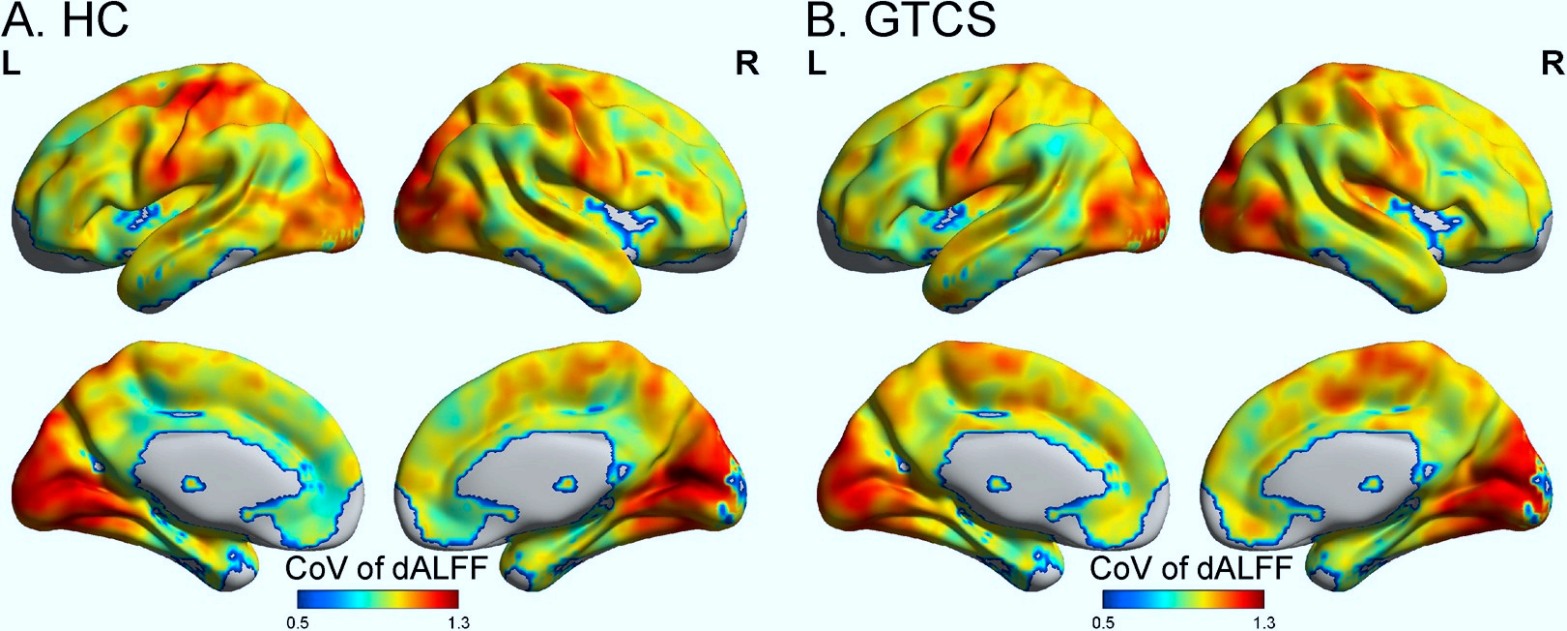
The pattern of temporal variability of the HC (A) and GTCS group (B). The temporal variability of dALFF was averaged at each voxel across all subjects in each group. Low and high variances of dALFF are shown in red and yellow colors, respectively. Abbreviations: HC, healthy control; GTCS, generalized tonic-clonic seizures; dALFF, amplitude of low-frequency fluctuation.

Temporal variability of dALFF was quantified for each voxel for the GTCS patients and HCs (Fig 1, S1 & S2 Files). The variance of dALFF exhibited a non-uniform spatial distribution. The largest temporal variability of dALFF was located in the heteromodal association cortex, whereas the lowest variability was located in the limbic cortex. Brain areas showing a moderate level of variability were the primary sensory and visual cortices, as well as regions upstream and downstream of the unimodal cortices. This spatial pattern of temporal variability of dALFF was consistent with previously reported results [24,25].

**Fig 2:**
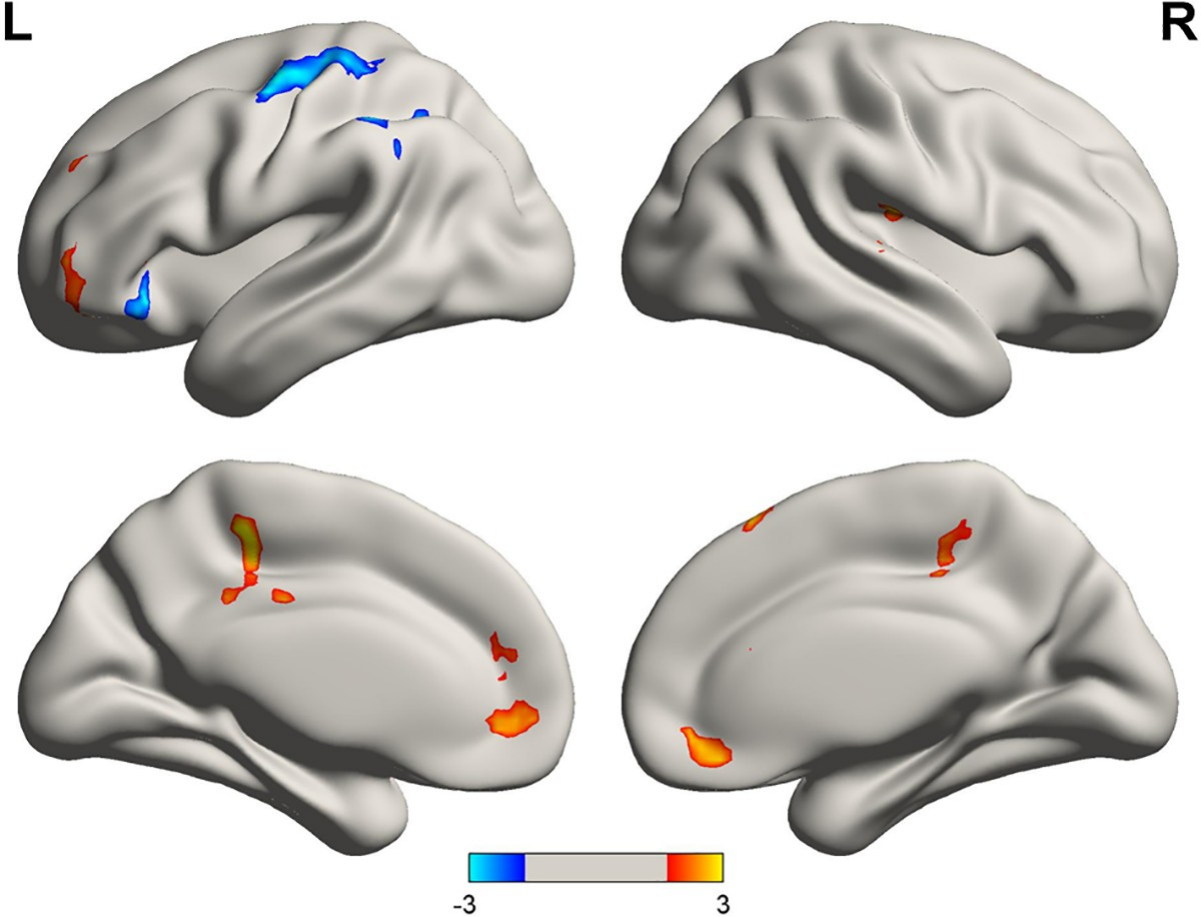
Group differences of temporal variability of the dALFF. The difference of temporal variability of dALFF between the GTCS and HC groups was identified using two-sample t-tests. Abbreviations: dALFF, amplitude of low-frequency fluctuation; GTCS, generalized tonic-clonic seizures; HC, healthy control.

The difference between dALFFs in the GTCS patients and HCs was compared using a two-sample t-test (Fig 2). Compared to HCs, the patient group showed increased temporal variability of ALFF in the subgenual (sg) ACC, superior frontal gyrus (SFG), middle cingulate cortex (MCC), medial frontal gyrus (MFG), right Rolandic operculum (ROL), and the left orbital portion of the inferior frontal gyrus. Temporal variability of ALFF was decreased in the left inferior parietal gyrus (IPG) and precentral gyrus (PreCG) in patients relative to HCs (Table 3).

**Table 3:** Brain regions showing abnormal temporal variability of dALFF (CoV of dALFF) in GTCS compared to HCs.

Brain region	Brodmann area	MNI coordinates (x, y, z)	Cluster size (voxels)	T-value
subgenual anterior cingulate cortex	10/32	(9, 42, –12)	151	3.13
Left orbitofrontal gyrus	10/11	(–36, 42, –12)	174	3.25
Right Rolandic operculum	40/41	(51, –18, 18)	174	3.44
Medial frontal gyrus	32	(6, 27, 57)	218	3.49
middle cingulate cortex	5/31	(–12, –33, 54)	169	4.17
Left inferior parietal lobule	40	(–51, –39, 42)	178	–3.08
Left precentral gyrus	3/4/6	(–42, –9, 57)	291	–3.44

### Relationship between dALFF and clinical variables

**Fig 3:**
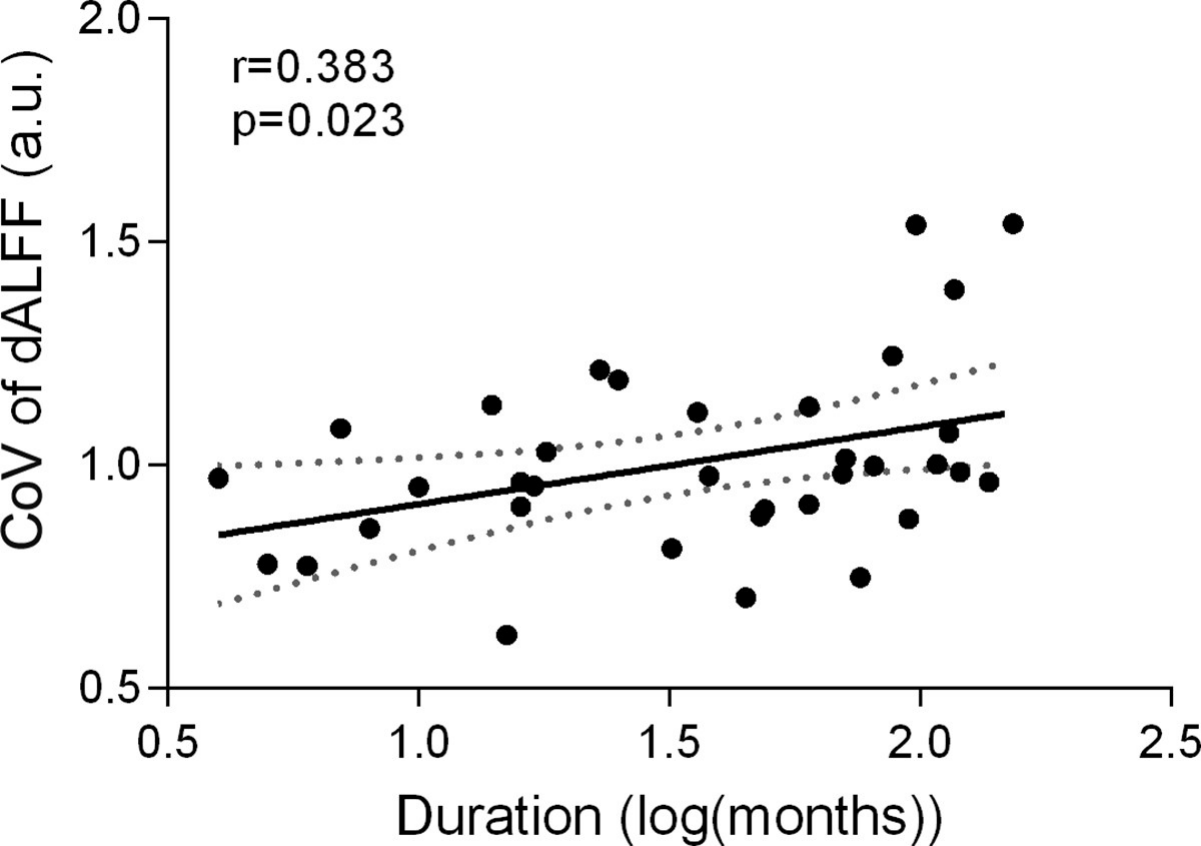
Temporal variability of dALFF in sgACC positively correlated with duration of disease. Abbreviations: dALFF, amplitude of low-frequency fluctuation; sgACC, subgenual anterior cingulate cortex.

There was a significant positive correlation between the duration of disease and dALFF in the sgACC (Fig 3). There was no significant correlation, however, between the duration of disease and dALFF in all other brain regions. In addition, there was no significant correlation between time of disease onset and dALFF in any brain region.

## Discussion

In the present study, we identify for the first time the dynamics of brain activity between patients with GTCS and HCs using a novel method to measure temporal variability of dALFF. We found that patients with GTCS exhibited increased temporal variability of dALFF, mainly in the sgACC, SFG, and MCC–areas involved in the default mode network (DMN)–whereas decreased variability of dALFF was observed in the somatomotor cortex and IPL. Furthermore, temporal variability of dALFF in the sgACC was found to be positively correlated with the duration of disease.

To the best of our knowledge, there are no prior studies to have used temporal dynamics to detect interictal epileptic-related brain activity changes from fMRI data. However, the temporal dynamics of the epileptic network have widely investigated using EEG and magnetoencephalography (MEG) data sets [34–36]. Conventional ALFF can be considered a proxy for static brain activity, and several previous studies have reported that static brain activity is altered in various forms of epilepsy [37–39]. Specifically, patients with GTCS show increased static ALFF in the bilateral thalamus and putamen; and decreased static ALFF in the mesial prefrontal cortex, bilateral dorsolateral prefrontal cortices, and bilateral orbital frontal cortices [16]. However, ALFF only generates a single, static estimate of brain activity. Dynamic brain activity (temporal variability of dALFF) describes the temporal changes in energy consumption [24], which may be a consequence of higher demand [40]. This novel temporal variability may also serve as a signature able to be used for clinical characterization [24,41].

GSWD-related brain activation in the thalamus, along with de-activation in the DMN, have been previously reported, and are suggested to indicate the generation of seizures and the suspension of brain default function [42,43]. In the present study, we demonstrated that the dynamic of brain activity changes in GTCS, extending the static ALFF. However, the dALFF is a means of capturing the dynamics of brain activity patterns using sliding-widow, rather than full-length time series [40]. Dynamic brain activity, excessive variability (increased temporal variance), or excessive stability (decreased temporal variance) [44] may occur under different conditions at different times, and may in fact be the cause of altered cognitive functions and particular pathological states [24,28]. Thus, our results support the idea that brain default function abnormalities may be the underlying pathophysiological cause of GTCS. Moreover, our results suggest that dALFF measurement may be a promising alternative approach to other existing methods for fMRI investigation into epilepsy.

Patients with GTCS showed decreased brain activity in the cortices [45,46], resulting in either synchronization or inhibition of cortical activity due to thalamocortical interactions [47,48]. Clinically, these interactions would result in a brief impairment of consciousness accompanied by minimal somatosensory signs [49,50]. For patients with GTCS, muscle rigidity and muscle contractions are seen during seizures [6], which are thought to contribute to abnormalities in the motor and sensory-thalamic systems [6]. The current findings provide additional evidence for abnormal brain dynamics of somatosensory networks in GTCS.

There exist several limitations to the current study. First, the number of subjects was relatively small. Second, the heterogeneity of the patients included in the present study is strongly suggested by the different antiepileptic drugs or combination of antiepileptic drugs. The future works should minimize this effect by collect the homogenous patients. Moreover, we could not investigate the effects of antiepileptic drugs, which are known to affect normal neuronal function, and in some cases produce cognitive impairments [51]. Third, we did not collect the heart rate and respiratory traces during fMRI scanning. These respiratory and cardiac fluctuations may reduce the specificity of low-frequency fluctuations in brain activity using a low-pass filter [28]. Fourth, the BOLD signal in WM has unique biological underpinnings [52], and has been widely used as a measure for clinical research [53]. There are no available studies investigating the effect of WM regression on dALFF analysis. In line with previous studies [24,53], we chose to regress out the WM signal in preprocessing. Finally, we did not evaluate the effects of GSWDs on brain dynamics, since simultaneous EEG data were not acquired.

## Conclusion

Patients with GTCS showed hyper-temporal variability of dALFF in parts of the DMN, and hypo-temporal variability in the somatomotor cortex, suggesting both an excessive variability and excessive stability in GTCS. Importantly, our results confirm that dynamic spontaneous brain activity contributes to the pathophysiological mechanisms of generalized seizures.

## Supporting information

S1 File The group mean dynamic ALFF pattern of the HC is included in ‘dALFF_meanHC.nii’. (RAR)

S2 File The group mean dynamic ALFF pattern of the patients of GTCS is included in ‘dALFF_meanGTCS.nii’. (RAR)
